# Safety and efficiency of deep brain stimulation in the elderly patients with Parkinson's disease

**DOI:** 10.1111/cns.14899

**Published:** 2024-08-06

**Authors:** Weidong Wu, Shun Gong, Shimiao Wang, Wei Lei, Lijia Yuan, Wei Wu, Jiqing Qiu, Weijin Sun, Guoming Luan, Minwei Zhu, Xudong Wang, Guobiao Liang, Yingqun Tao

**Affiliations:** ^1^ Department of Neurosurgery General Hospital of Northern Theater Command Shenyang China; ^2^ China Medical University Shenyang China; ^3^ Department of Neurosurgery First Hospital of Jilin University Changchun China; ^4^ Department of Neurosurgery, Sanbo Brain Hospital Capital Medical University Beijing China; ^5^ Department of Neurosurgery First Affiliated Hospital of Harbin Medical University Harbin China

**Keywords:** deep brain stimulation, elderly, neurosurgical robot, Parkinson's disease

## Abstract

**Aims:**

Deep brain stimulation (DBS) is not routinely performed in elderly patients (≥75 years old) to date because of concerns about complications and decreased benefit. This study aimed to evaluate the safety and efficacy of DBS in elderly patients with Parkinson's disease.

**Methods:**

A retrospective analysis was performed using data from 40 elderly patients from four centers who were treated with neurosurgical robot‐assisted DBS between September 2016 and December 2021. These patients were followed up for a minimum period of 2 years, with a subgroup of nine patients followed up for 5–7 years. Patient demographic characteristics, surgical information, pre‐ and postoperative motor scores, non‐motor scores, activities of daily living, and complications were retrospectively analyzed.

**Results:**

The mean surgical procedure duration was 1.65 ± 0.24 h, with a mean electrode implantation duration of 1.10 ± 0.23 h and a mean pulse generator implantation duration of 0.55 ± 0.07 h. The mean pneumocephalus volume, electrode fusion error, and Tao's DBS surgery scale were 16.23 ± 12.81 cm^3^, 0.81 ± 0.23 mm, and 77.63 ± 8.08, respectively. One patient developed a skin infection, and the device was removed. The Unified Parkinson's disease rating scale, Unified Parkinson's disease rating scale of Part III, tremor, rigidity, bradykinesia, axial, and Barthel index for activities of daily living (ADL‐Barthel) scores significantly improved at the 2‐year follow‐up (*p* < 0.05). The levodopa equivalent daily dose (LEDD) was significantly reduced at the 2‐year follow‐up (*p* < 0.05). However, the Montreal cognitive assessment, Hamilton depression scale, and Hamilton anxiety scale scores did not significantly change during the 2‐year follow‐up (*p* > 0.05). Additionally, in the subgroup with a 5‐year follow‐up, the motor symptoms, ADL‐Barthel score, and cognitive function worsened over time compared to baseline. However, there was still an improvement in motor symptoms and ADL with DBS on‐stimulation compared with the off‐stimulation state. The LEDD increased 5 years after surgery compared to that at baseline. Eleven patients had passed away during follow‐up, the mean survival time was 38.3 ± 17.3 months after surgery, and the mean age at the time of death was 81.2 (range 75–87) years.

**Conclusion:**

Robot‐assisted DBS surgery for the elderly patients with Parkinson's disease is accurate and safe. Motor symptoms and ADL significantly improve and patients can benefit from long‐term neuromodulation, which may decrease the risk of death.

## Graphical abstract

Robot‐assisted DBS surgery for the elderly PD patients is accurate and safe. The motor symptoms and ADL are significantly improved and can benefit from neuromodulation for long term, which may decline the risk of death.

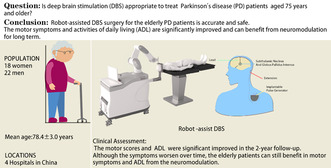

## 1 INTRODUCTION

Deep brain stimulation (DBS) is a mature and effective technology that improves the motor symptoms, some of the non‐motor symptoms, and quality of life for patients with Parkinson's disease (PD) by implanting stimulating electrodes in the brain.^1^ However, DBS for elderly patients (≥75 years) with PD still remains controversial because of concerns about complications and decreased benefit. As for the age limit of DBS, the guideline recommended consideration of overall health and biological age rather than numerical age,^2^ and the consensus of Chinese experts recommended that the age of patients is usually <75 years.^3^ Since 2014, the development of neurosurgical robots in the field of DBS has shown safety, accuracy, and efficiency, especially under general anesthesia in DBS surgery.^4^, ^5^, ^6^ Additionally, with the aging of the social population, there is a growing number of elderly individuals with PD. Consequently, our center is attempting to use neurosurgical robot‐assisted DBS in elderly patients with PD together with several centers that use neurosurgical robot‐assisted DBS in China, to evaluate the safety and efficacy of DBS in elderly patients with PD.

## 2 METHODS

### 2.1 Patients and study design

**FIGURE 1:**
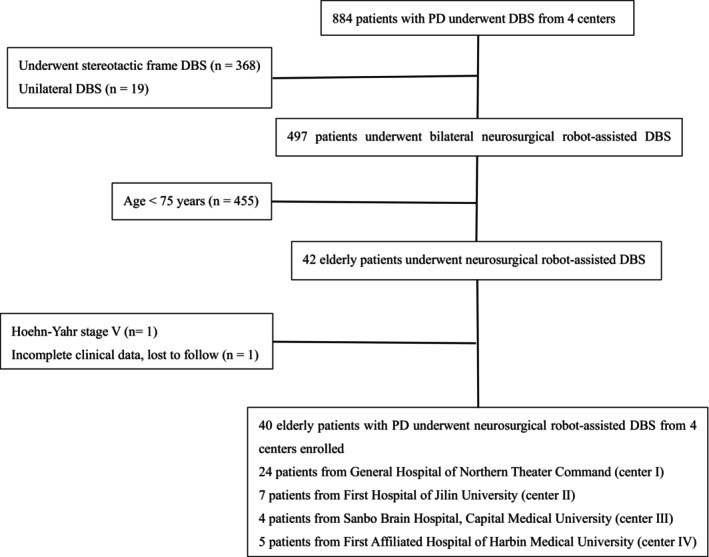
Flowchart of elderly patients who underwent neurosurgical robot‐assisted DBS selection. DBS, Deep brain stimulation; PD, Parkinson's disease.

Elderly patients (≥75 years) with PD who underwent neurosurgical robot‐assisted DBS between September 2016 and December 2021 were retrospectively analyzed. The study population included patients treated at four centers (Figure 1; center I: 24/40, 60%; center II: 7/40, 17.5%; center III: 4/40, 10%; and center IV: 5/40, 12.5%). Thirty‐one patients underwent bilateral subthalamic nucleus (STN) DBS, and nine underwent bilateral globus pallidus internus (GPi) DBS. The clinical diagnosis of PD was made according to the British PD Society Brain Bank criteria.^7^ This study was approved by the local Institutional Ethics Committee (Grant No. ChiCTR2200055850) and complied with the ethical principles of the Declaration of Helsinki. All the participants provided written informed consent.

The inclusion criterion was neurosurgical robot‐assisted DBS for PD. The exclusion criteria were as follows: (1) Hoehn–Yahr stage V, (2) age <75 years, (3) unilateral DBS surgery, and (4) incomplete clinical data.

### 2.2 Neurosurgical robot‐assisted DBS surgical procedures

All patients underwent head MRI and contrast‐enhanced CT before DBS surgery. If patients take antiplatelet medications preoperatively, the platelet aggregation rate must be tested. Surgery should proceed only once platelet aggregation rates and coagulation function return to normal. Before contrast‐enhanced CT scanning, four to six metal marker screws were fixed to each patient's skull. The MRI and CT images were imported into the ROSA (33 cases used the ROSA Robot of Surgery Assistant, Medtech S.A.S., France) or Remebot (seven cases used the Remebot Robot of Surgery Assistant, Beijing Baihui Weikang Technology Co., Ltd., China) software systems. In the ROSA software system, as metal artifacts of bone markers are present in CT images, the modified registration method was used in the ROSA‐assisted DBS surgery, which can reduce the registration error and electrode vector error, as published by our center.^8^ The surgical trajectory was designed according to the location of the nuclei and the best cortical puncture point, where the gyrus cortex was closest to the dura mater, simultaneously avoiding the sulci and blood vessels in images. Under general anesthesia, all patients underwent surgery in the supine position, with the head elevated by approximately 20°. The patient's head was tightly fixed to the robot's connecting rod. The burr hole was opened using a drill and a parallel steel cannula was inserted after opening the dura mater. Microelectrode recording (MER) was performed intraoperatively using an alpha‐omega microelectrode recording system to confirm that the target was correct. The burr hole was closed with bone wax, and the lead was fixed after the steel cannula was pushed out following the implantation of the electrode (four electrodes of Medtronic 3389 and 12 electrodes of Medtronic 3387 [Medtronic, Ltd., Minneapolis, MN, USA]; 58 PINS L‐301 electrodes and six PINS L‐302 electrodes [Beijing PINS Medical Co., Beijing, China]). Then, bilateral implantable pulse generators (IPG) were implanted into the subcutaneous pockets of the infraclavicular region (19 patients used un‐rechargeable IPG and 21 patients used rechargeable IPG).

The initial neuromodulation was performed 3–4 weeks after surgery using Toronto Western Hospital algorithms or a modified power‐on programming method that chooses the stimulation contact depending on the typical intraoperative MER signal and the electrode contact position for DBS programming for PD.^9^, ^10^ The follow‐up examinations for neuromodulation were usually every 6 months or when the symptoms deteriorated. The DBS power‐on voltage and adverse events during neuromodulation were recorded at 6‐, 12‐, and 24‐month follow‐up examinations.

### 2.3 Postoperative assessment

The duration of the surgical procedure (electrode implantation duration + pulse generator implantation duration), electrode implantation duration,^11^ pneumocephalus volume at 2 h post‐operation,^11^ electrode fusion error at 1 week postoperatively,^5^ Tao's DBS surgical scale,^11^ and perioperative complications were evaluated.

### 2.4 Clinical follow‐up and assessment

During neuromodulation and follow‐up, the Unified Parkinson's disease rating scales (UPDRS), the Unified Parkinson's disease rating scale of Part III (UPDRS‐III), tremor score (UPDRS item 20, 21), rigidity score (UPDRS item 22), bradykinesia score (UPDRS item 23, 24, 25, 26, 31), axial score (UPDRS items 27, 28, 29, 30), levodopa equivalent daily dose (LEDD), and Barthel index for activities of daily living (ADL‐Barthel) were assessed in the off medication/on stimulation state. Cognition was evaluated using the Montreal cognitive assessment (MoCA), and emotion was assessed using the Hamilton depression scale (HAMD) and Hamilton anxiety scale (HAMA) in the on‐medication/on‐stimulation state. For a subset of nine of these patients, follow‐up data beyond 5 years were available in both off‐medication/off‐stimulation and off‐medication/on‐stimulation states. Off‐medication was defined as a 12‐h overnight withdrawal of antiparkinsonian medication. Clinical evaluation was conducted by two raters from four centers. Data on the deceased patients were collected during the follow‐up period until December 2023.

### 2.5 Statistical analysis

Descriptive statistics were used to analyze the baseline characteristics. Data comparing baseline and postoperative scores were analyzed using the Student's *t*‐test if the data conformed to a normal distribution; otherwise, the Wilcoxon signed‐rank test was used. Analyses were conducted using SPSS Statistics Desktop (version 22.0; IBM Corp.). Statistical significance was set at *p* < 0.05 (two‐sided).

$$\text{Improvement rate}=\frac{|\text{Postoperative score}-\text{Preoperative score}|}{\text{Preoperative score}}$$

## 3 RESULTS

Four centers admitted 884 patients with PD who underwent DBS. Among these patients, 368 were excluded because of treatment with stereotactic frame DBS, 19 were treated with unilateral DBS, and 497 underwent bilateral neurosurgical robot‐assisted DBS. Further application of the exclusion criteria resulted in the exclusion of 455 patients aged <75 years, one case of Hoehn–Yahr stage V, and one case with incomplete clinical data. The final cohort consisted of 40 elderly patients who underwent neurosurgical robot‐assisted DBS. (Figure 1).

### 3.1 Baseline of patient clinical characteristics

Of the 40 elderly patients with PD who underwent neurosurgical robot‐assisted DBS included in this study, two died and could not be evaluated in 2 years. The study cohort included 22 males (55%). The mean age at DBS surgery was 78.4 ± 3.0 years. The disease duration was 10.2 ± 5.6 years, and Hoehn–Yahr stage was 3.4 ± 0.5. Levodopa response of UPDRS improvement 29.5 ± 5.7%, UPDRS‐III improvement 38.0 ± 5.2%. The clinical characteristics of the patients are shown in Table 1 and Table S1 (patients are sorted according to surgical date).

**TABLE 1:** The baseline of elderly patients with Parkinson's disease.

Characteristic	Value
Age (years)	
Mean	78.4 ± 3.0
Range	75–86
Sex (no. of patients)	
Male	22
Female	18
Hoehn–Yahr state	3.4 ± 0.5
Duration of disease (years)	10.2 ± 5.6
Basic disease (no. of patients)	
Hypertension	11
Diabetes	5
Coronary disease	9
Tumor History	1
Main motor symptoms (no. of patients)	
Tremor	18
Rigidity	2
Bradykinesia	20
Levodopa response of UPDRS‐III improvement (%)	38.0 ± 5.2
Target (Bilateral) (no. of patients)	
STN	31
GPi	9
Lead Model (no. of electrodes)	
PINS L301	58
PINS L302	6
Medtronic 3387	12
Medtronic 3389	4
IPG type (no. of patients)	
Rechargeable	21
Un‐rechargeable	19 (STN‐DBS:18 cases; GPi‐DBS:1 case)

*Note*: Plus–minus values are means ±SD.

Abbreviations: DBS, Deep brain stimulation; GPi, Globus pallidus internus; IPG, Implantable pulse generator; STN, Subthalamic nucleus; UPDRS‐III, the Unified Parkinson's disease rating scale of Part III.

### 3.2 Postoperative outcome and neuromodulation

The surgical procedure duration was 1.65 ± 0.24 h, with an electrode implantation duration of 1.10 ± 0.23 h and a pulse generator implantation duration of 0.55 ± 0.07 h. The pneumocephalus volume, electrode fusion error, and Tao's DBS surgical scale were 16.23 ± 12.81 cm^3^, 0.81 ± 0.23 mm, and 77.63 ± 8.09. One patient (case 30) developed an infection at the incision behind the ear 30 months after surgery. Consequently, the entire DBS system, comprising the electrodes, leads, and pulse generator, was completely removed. No other complications, such as intracranial hemorrhage, occurred in our group. The stimulation parameters of amplitude, frequency, and pulse width used for DBS are listed in Table S2. The postoperative side effects during neuromodulation at 6, 12, and 24‐month were dizziness (*n* = 65), muscle contraction (*n* = 9), oculomotor dysfunction (*n* = 23), palpitations (*n* = 2), and dyskinesia (*n* = 39). These side effects were alleviated by adjusting the stimulation parameters.

### 3.3 Clinical assessment during follow‐up

With stimulation in the medication‐off state, the UPDRS improved from the baseline value of 58.53 ± 11.19 by 39% at 1 year and 24% at 2 years. The UPDRS‐III improved from the baseline value of 27.55 ± 5.61 by 43% at 1 year and 27% at 2 years. Compared to baseline, the scores for tremor improved by 69% and 55%, rigidity by 45% and 23%, bradykinesia by 42% and 26%, and axis scores by 34% and 21% at 1 year and 2 years, respectively. LEDD was reduced by 49% at 1‐year and 34% at 2‐year. The ADL‐Barthel scores improved by 63% at 1 year and 42% at 2 years compared to baseline. The MoCA, HAMD, and HAMA showed no significant changes during the 2‐year follow‐up. (Table 2).

**TABLE 2:** Clinical assessment during the follow‐up (off medication/on stimulation).

Item	Baseline (*n* = 40)	1‐year FU (*n* = 39)	2‐year FU (*n* = 38)	*p* Value (1‐year vs. baseline)	*p* Value (2‐yeaar vs. baseline)
UPDRS	58.53 ± 11.19	35.77 ± 10.47	44.32 ± 13.48	<0.001	<0.001
UPDRS‐III	27.55 ± 5.61	15.69 ± 4.69	20.13 ± 4.75	<0.001	<0.001
Tremor	3.85 ± 1.89	1.18 ± 0.88	1.74 ± 0.92	<0.001	<0.001
Rigidity	2.05 ± 0.96	1.13 ± 0.66	1.58 ± 0.60	<0.001	0.023
Bradykinesia	9.73 ± 2.81	5.69 ± 2.13	7.24 ± 2.36	<0.001	<0.001
Axis score	7.23 ± 2.53	4.77 ± 2.05	5.74 ± 1.95	<0.001	0.031
LEDD	628.61 ± 231.63	323.72 ± 208.37	414.47 ± 214.09	<0.001	<0.001
ADL‐Barthel	50.38 ± 16.23	81.92 ± 11.62	71.58 ± 12.90	<0.001	<0.001
MoCA^a^	21.18 ± 2.48	21.08 ± 2.45	20.89 ± 2.42	0.933	0.632
HAMD^a^	15.70 ± 4.27	15.59 ± 4.71	15.71 ± 4.82	0.913	0.992
HAMA^a^	14.73 ± 4.42	14.64 ± 4.64	14.95 ± 4.70	0.809	0.876

*Note*: Values are shown as mean ± SD.

Abbreviations: ADL‐Barthel, Barthel index for activities of daily living; FU, Follow‐up; HAMA, Hamilton anxiety scale; HAMD, Hamilton depression scale; LEDD, the levodopa equivalent daily dose; MoCA, Montreal cognitive assessment; UPDRS, the Unified Parkinson's disease rating scales; UPDRS‐III, the Unified Parkinson's disease rating scale of part III.

^a^ MoCA, HAMD and HAMA were evaluated in the on medication/on stimulation state.

**FIGURE 2:**
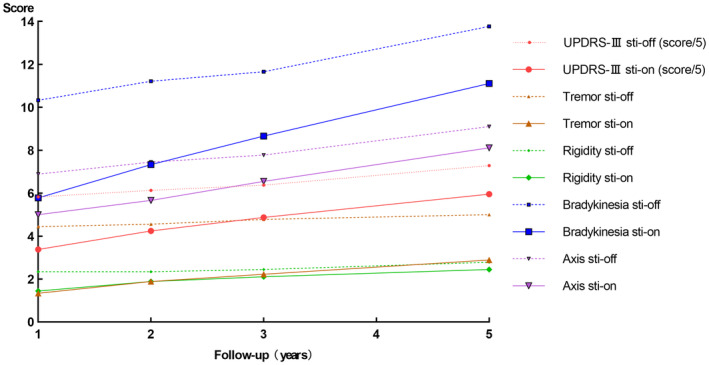
Clinical assessment of subgroup of 5‐year follow‐up (off‐medication, *n* = 9). ADL‐Barthel, Barthel index for activities of daily living; Sti‐off, stimulation off; Sti‐on, stimulation on; UPDRS‐III, Unified Parkinson's disease rating scale of Part III.

In the 5‐year follow‐up subgroup, motor symptoms and the ADL‐Barthel score improved in the early postoperative period and then gradually worsened between 1‐year and 5‐year. However, when compared with the off‐medication/off‐stimulation state at the 5‐year follow‐up, the scores for UPDRS‐III improved by 18.3%, tremor by 42.2%, rigidity by 12.2%, bradykinesia by 19.4%, axis by 11%, and ADL‐Barthel by 47.3% in the off‐medication/on‐stimulation state. The LEDD decreased after surgery but gradually increased between the 1‐year and 5‐year follow‐ups, rising from a baseline of 525.02 mg and increasing to 558.33 mg at the 5‐year follow‐up. The MoCA declined during the long‐term follow‐up and deteriorated by 14.7% at the 5‐year follow‐up compared with baseline (Figure 2 and Table S3).

### 3.4 Survival status during follow‐up

The mean follow‐up was 46.3 ± 21.7 (range 24.5–88.6) months. Nineteen patients (18 cases of STN‐DBS and one case of GPi‐DBS) used un‐rechargeable IPG, and three patients of STN‐DBS underwent replacing IPG surgery (one patient at 79.3 months and two patients at 69.6 months after DBS surgery). During the follow‐up, a total of 11 patients had passed away, comprising seven males and four females. The mean age at the time of surgery was 78.0 ± 2.3 (range 75–81) years, and the mean survival time was 38.3 ± 17.3 months after the operation, the mean age at the time of death was 81.2 ± 3.2 (range 75–87) years. Among the causes of death, four patients (cases 2, 9, 12, and 13) died due to the progression of PD; three patients (cases 4, 14, and 24) died of severe pneumonia; two patients (cases 5 and 32) died of cardiovascular disease; one patient (case 10) died of lung cancer; and one patient (case 22) died of postoperative anorexia (Table 3).

**TABLE 3:** The deceased of the elderly patients with Parkinson's disease.

No.	Age at surgery (years)	Age at death (years)	Survival time after DBS surgery (months)	Cause of death
2	76	80	49.4	Progress of Parkinson's disease
4	77	81	48.3	Pneumonia
5	81	87	70.0	Cardiovascular diseases
9	81	83	25.5	Progress of Parkinson's disease
10	79	82	35.2	Lung cancer
12	76	80	47.8	Progress of Parkinson's disease
13	81	84	35.9	Progress of Parkinson's disease
14	79	83	51.6	Pneumonia
22	75	75	4.7	Postoperative anorexia
24	76	79	33.2	Pneumonia
32	77	79	19.7	Cardiovascular diseases

Abbreviations: DBS, Deep brain stimulation; No., Number, sort by surgical date, matching the number in Table S1.

## 4 DISCUSSION

To our knowledge, this is the most reported case of a multicenter experience involving 40 elderly patients (≥75 years) with PD who underwent neurosurgical robot‐assisted DBS. Although the age limit for DBS remains controversial, advancements in neurosurgical robot‐assisted DBS are being considered for elderly individuals with PD after careful assessment. Our results suggest that robot‐assisted neurosurgical DBS is a safe and effective treatment for elderly patients with PD.

The effectiveness of DBS largely depends on the accuracy of electrode implantation. Evidence indicates that brain atrophy in the elderly patients will increase the pneumocephalus volume during the DBS surgery, which can decline the accuracy of electrode implantation.^12^ We found that the pneumocephalus volume (16.23 ± 12.81 cm^3^) in 40 elderly patients was larger than our previous report (8.30 cm^3^); however, the electrode fusion error (0.81 ± 0.23 mm) was similar to our previous report (0.74 mm).^5^ This may be attributed to the use of robot‐assisted surgery, in which the electrode error was smaller than in stereotactic frame surgery.^13^ Additionally, other studies also report that robot‐assisted guided DBS is accurate and precise for lead implantation.^14^, ^15^ The robot‐guided DBS obviates the need for checking and switching coordinates, as is standard for frame‐based DBS, reduces the chance for human error, and shortens operation time, which reduces cerebrospinal fluid loss and enhances accuracy. Previous studies on DBS in the elderly have reported a few surgical complications.^16^, ^17^ In our study, only one patient had an infection 30 months after surgery. No other complications, such as intracranial hemorrhage, occurred in our group. This may be due to individual factors in surgical complications, and the incidence of surgical complications in elderly patients has not increased.^18^, ^19^ Furthermore, asleep robot‐assisted DBS with a proficient surgical team makes patients more comfortable during surgery, alleviates intraoperative blood pressure fluctuations, and shortens surgical time, which may reduce the risk of bleeding and infection. Consequently, asleep robot‐assisted DBS treatment in elderly patients with PD is accurate, safe, and efficient.

Literature on the clinical outcomes of DBS in patients aged ≥75 years is limited. Sharma et al. found elderly patients (*n* = 30) who underwent DBS had motor scores improved by 27.3% after a mean follow‐up of 2.5 years.^16^ Another study reported motor scores improved by 45.7% at 1 year and 8.6% at a mean follow‐up of 55.08 months in elderly patients (*n* = 27) after DBS.^17^ In our study, elderly patients who underwent DBS showed significant improvements in motor scores (27%), tremor (55%), rigidity (23%), bradykinesia (26%), and axial score (21%) at the 2‐year follow‐up, similar to previous reports. Meanwhile, the ADL‐Barthel score improved by 63% at 1‐year and 42% at 2‐year of follow‐up. However, there is a lack of evidence in the literature to support the choice between STN or GPi DBS in elderly patients.^20^ In our study, we also focused on the patient's imaging features. When severe nuclear atrophy, calcification, or infarction occurs within the nucleus, another target is selected as the primary surgical target. In the 5‐year follow‐up subgroup, our results showed that motor symptoms, ADL, and cognitive function worsened over time compared to baseline. Nevertheless, improvement in motor symptoms and ADL with DBS on‐stimulation compared with the off‐stimulation state was still observed. Meanwhile, LEDD gradually increased during follow‐up, with some patients requiring antipsychotic drugs. This may be attributed to a decrease in therapeutic response with PD progression and an association with age‐related comorbidities.

The life expectancy at birth in mainland China was 77.7 years old in 2019.^21^ Weaver et al. found patients with PD who underwent DBS had longer survival days than those who did not receive DBS.^22^ In our study, 11 patients had passed away, and the mean age at the time of death was 81.2 years, which was higher than the life expectancy in China. The causes of death were mostly progressive PD and cardiopulmonary disease. Although DBS does not delay the progression of PD, it can enhance the quality of life by improving motor symptoms and ADL, which may reduce the risk of death in elderly patients with PD. Moreover, only three patients replaced the IPG among the 18 patients who underwent STN‐DBS with an un‐rechargeable IPG. Considering the mean survival time of 38.3 ± 17.3 months post‐operation, choosing an un‐rechargeable IPG for STN‐DBS may be more cost‐effective for elderly patients and avoid the potential serious consequences of forgetting to recharge the IPG.

This study has several limitations. First, although this was a multicenter retrospective study, it included a limited number of patients. However, this is the largest number of case reports on elderly patients (≥75 years) with PD who underwent neurosurgical robot‐assisted DBS. Second, our study, which included different robot designs, electrode models, and DBS targets, may have introduced a bias in the results. Third, patients were not evaluated using all neuropsychological tests at every follow‐up visit because of the progression of PD, as some patients were unable to cooperate in completing the examination.

## 5 CONCLUSION

Our results suggest that neurosurgical robot‐assisted DBS is accurate, safe, and efficient, making it suitable for elderly patients with PD. Although symptoms worsen over time, elderly patients can still benefit from neuromodulation for motor symptoms and ADL, which may decrease the risk of death.

## AUTHOR CONTRIBUTIONS

Shimiao Wang, Wei Lei, Lijia Yuan, Wei Wu, Jiqing Qiu, Weijin Sun, Yuguang Guan, Minwei Zhu, and Xudong Wang collected the data. Jiqing Qiu, Yuguang Guan, Minwei Zhu, and Yingqun Tao performed surgery. Weidong Wu, Shun Gong, and Yingqun Tao performed all the statistical analyses. Weidong Wu and Shun Gong drafted the manuscript. Guobiao Liang critically revised the manuscript. Yingqun Tao designed the study and critically revised the manuscript.

## FUNDING INFORMATION

This study was supported by grants from the National Natural Science Foundation of China (81870890), and the Applied Basic Research Program of Liaoning, China (2022JH2/101300055; 2023JH2/101700104).

## CONFLICT OF INTEREST STATEMENT

None.

## Supporting information


Table S1.

Table S2.

Table S3.

## Data Availability

Data are available upon reasonable request to the corresponding author. The data that supports the findings of this study are available in the supplementary material of this article.
