# Supplementary material for: Safety and efficiency of deep brain stimulation in the elderly patients with Parkinson's disease
Source: CNS Neurosci Ther. 2024 Aug 6;30(8):e14899. doi: 10.1111/cns.14899 (PMC11303456; doi:10.1111/cns.14899)
Supplement: Supplementary file 1 — Table S1. [file CNS-30-e14899-s001.docx]

Supplemental Table 1. The baseline of elderly patients with Parkinson’s disease (Sort by surgical date)

| NO. | Age | Sex | Duration (years) | Hoehn-Yahr state | Basic disease | Main motor symptoms | Levodopa response of UPDRS-Ⅲ improvement (%) | Target (bilateral) | Survival situation |
| --- | --- | --- | --- | --- | --- | --- | --- | --- | --- |
| 1 | 75 | M | 14 | 3 | Hypertension | Tremor | 46.1 | STN | Alive |
| 2 | 76 | M | 14 | 4 | Hypertension | Tremor | 41.2 | STN | Deceased |
| 3 | 76 | F | 2 | 2.5 | Coronary disease | Tremor | 35.4 | STN | Alive |
| 4 | 77 | M | 17 | 4 | / | Tremor | 36.3 | STN | Deceased |
| 5 | 81 | F | 5 | 4 | / | Rigidity | 32.5 | STN | Deceased |
| 6 | 80 | M | 6 | 3 | Hypertension | Bradykinesia | 30.0 | STN | Alive |
| 7 | 85 | F | 8 | 4 | Coronary disease, Gastric cancer resection | Bradykinesia | 32.0 | STN | Alive |
| 8 | 86 | M | 6 | 4 | Hypertension, Coronary disease | Bradykinesia | 33.3 | STN | Alive |
| 9 | 81 | M | 5 | 4 | / | Tremor | 33.3 | STN | Deceased |
| 10 | 79 | F | 12 | 4 | / | Tremor | 38.2 | STN | Deceased |
| 11 | 76 | F | 7 | 3 | / | Tremor | 38.7 | STN | Alive |
| 12 | 76 | F | 20 | 4 | / | Tremor | 46.9 | STN | Deceased |
| 13 | 81 | M | 20 | 3 | / | Tremor | 45.0 | GPI | Deceased |
| 14 | 79 | M | 11 | 4 | / | Bradykinesia | 45.2 | GPI | Deceased |
| 15 | 79 | F | 5 | 3 | Hypertension, Diabetes | Tremor | 31.2 | GPI | Alive |
| 16 | 75 | M | 19 | 3 | Hypertension, Diabetes, Coronary disease | Bradykinesia | 44.8 | GPI | Alive |
| 17 | 78 | M | 13 | 4 | Hypertension | Bradykinesia | 47.3 | STN | Alive |
| 18 | 76 | M | 3 | 3 | Coronary disease | Bradykinesia | 33.6 | STN | Alive |
| 19 | 78 | F | 2 | 2.5 | / | Tremor | 35.4 | STN | Alive |
| 20 | 76 | F | 12 | 3 | / | Bradykinesia | 40.3 | STN | Alive |
| 21 | 81 | F | 16 | 3 | Coronary disease | Bradykinesia | 35.8 | STN | Alive |
| 22 | 75 | F | 9 | 3 | / | Tremor | 31.1 | GPI | Deceased |
| 23 | 76 | F | 8 | 3 | / | Tremor | 45.2 | GPI | Alive |
| 24 | 76 | M | 15 | 4 | / | Tremor | 32.8 | GPI | Deceased |
| 25 | 75 | M | 8 | 3 | / | Tremor | 38.6 | STN | Alive |
| 26 | 78 | M | 6 | 3 | Hypertension, Coronary disease | Bradykinesia | 35.4 | STN | Alive |
| 27 | 81 | M | 7 | 3 | / | Tremor | 31.3 | STN | Alive |
| 28 | 80 | F | 6 | 4 | / | Tremor | 40.0 | STN | Alive |
| 29 | 77 | F | 6 | 3 | Hypertension | Bradykinesia | 38.5 | STN | Alive |
| 30 | 80 | F | 10 | 3 | / | Bradykinesia | 35.4 | GPI | Alive |
| 31 | 77 | M | 7 | 3 | Hypertension | Bradykinesia | 38.6 | STN | Alive |
| 32 | 77 | M | 4 | 3 | Diabetes | Bradykinesia | 46.9 | GPI | Deceased |
| 33 | 82 | M | 18 | 3 | / | Tremor | 37.0 | STN | Alive |
| 34 | 80 | M | 6 | 3 | / | Bradykinesia | 37.0 | STN | Alive |
| 35 | 86 | M | 20 | 4 | / | Rigidity | 35.0 | STN | Alive |
| 36 | 78 | M | 12 | 4 | Diabetes, Coronary disease | Bradykinesia | 38.1 | STN | Alive |
| 37 | 78 | M | 12 | 4 | / | Bradykinesia | 39.2 | STN | Alive |
| 38 | 76 | F | 3 | 3 | Diabetes, Coronary disease | Bradykinesia | 43.2 | STN | Alive |
| 39 | 76 | F | 20 | 4 | Hypertension | Bradykinesia | 30.7 | STN | Alive |
| 40 | 75 | F | 15 | 3 | / | Bradykinesia | 42.7 | STN | Alive |

Abbreviations: NO., Number, sort by surgical date; M, Male; F, Female; UPDRS-Ⅲ, the Unified Parkinson’s Disease Rating Scale of part Ⅲ; STN, Subthalamic nucleus; GPi, Globus pallidus internus.
