# Supplementary material for: Safety and efficiency of deep brain stimulation in the elderly patients with Parkinson's disease
Source: CNS Neurosci Ther. 2024 Aug 6;30(8):e14899. doi: 10.1111/cns.14899 (PMC11303456; doi:10.1111/cns.14899)
Supplement: Supplementary file 2 — Table S2. [file CNS-30-e14899-s002.docx]

Supplemental Table 2. The stimulation parameters at 6-, 12-, and 24-month follow-up

| DBS Targets | Characteristic | 6-month FU (n=39,STN=31,GPi=8) | 12-month FU (n=39,STN=31,GPi=8) | 24-month FU (n=38,STN=31,GPi=7) |
| --- | --- | --- | --- | --- |
| STN | Mean amplitude, V | 2.31±0.44 | 2.61±0.45 | 2.79±0.50 |
|  | Mean frequency, Hz | 133.9±23.1 | 135.3±18.9 | 135.2±22.9 |
|  | Mean pulse width, ms | 59.0±7.2 | 62.3±9.3 | 63.2±9.2 |
| GPi | Mean amplitude, V | 2.96±0.53 | 3.31±0.43 | 3.65±0.55 |
|  | Mean frequency, Hz | 147.5±28.2 | 141.3±38.5 | 144.3±35.5 |
|  | Mean pulse width, ms | 67.5±8.6 | 71.9±7.5 | 75.0±9.4 |

Abbreviations: FU, Follow-up; DBS, Deep brain stimulation; STN, Subthalamic nucleus; GPi, Globus pallidus internus.

Values are shown as mean ± SD.
