# Supplementary material for: Safety and efficiency of deep brain stimulation in the elderly patients with Parkinson's disease
Source: CNS Neurosci Ther. 2024 Aug 6;30(8):e14899. doi: 10.1111/cns.14899 (PMC11303456; doi:10.1111/cns.14899)
Supplement: Supplementary file 3 — Table S3. [file CNS-30-e14899-s003.docx]

Supplemental Table 3. Clinical assessment of subgroup of 5-year follow-up (off medication, n=9)

| Item |  | Baseline | 1-year FU | 2-year FU | 3-year FU | 5-year FU |
| --- | --- | --- | --- | --- | --- | --- |
| UPDRS-Ⅲ | sti-on | 28.67±5.52 | 16.89±4.76 | 21.22±3.99 | 24.33±5.32 | 29.78±8.42 |
|  | sti-off |  | 29.11±5.11 | 30.67±4.50 | 31.89±4.57 | 36.44±7.78 |
| Tremor | sti-on | 4.33±1.87 | 1.33±0.87 | 1.89±0.93 | 2.22±0.97 | 2.89±1.17 |
|  | sti-off |  | 4.44±1.94 | 4.56±2.07 | 4.78±1.79 | 5.00±1.73 |
| Rigidity | sti-on | 2.33±0.71 | 1.44±0.53 | 1.89±0.33 | 2.11±0.33 | 2.44±0.73 |
|  | sti-off |  | 2.33±0.71 | 2.33±0.71 | 2.44±0.53 | 2.78±0.67 |
| Bradykinesia | sti-on | 9.89±2.98 | 5.78±1.86 | 7.33±1.73 | 8.67±2.55 | 11.11±4.08 |
|  | sti-off |  | 10.33±2.83 | 11.22±2.86 | 11.67±2.69 | 13.78±3.60 |
| Axis score | sti-on | 7.00±2.74 | 5.00±2.69 | 5.67±2.50 | 6.56±2.60 | 8.11±3.69 |
|  | sti-off |  | 6.89±2.85 | 7.44±2.51 | 7.78±2.59 | 9.11±3.82 |
| ADL-Barthel | sti-on | 47.78±17.16 | 78.89±12.19 | 69.44±10.14 | 59.44±16.09 | 45.00±21.36 |
|  | sti-off |  | 44.44±15.90 | 40.00±14.79 | 37.22±15.02 | 30.56±14.24 |
| LEDD |  | 525.02±164.90 | 288.89±208.83 | 427.78±246.36 | 469.44±238.74 | 558.33±255.56 |
| ^*^MoCA |  | 21.33±2.74 | 21.22±2.82 | 21.00±2.74 | 19.86±2.79; n=7 | 18.20±2.95; n=5 |

Abbreviations: FU, Follow-up; UPDRS-Ⅲ, the Unified Parkinson’s Disease Rating Scale of part Ⅲ; ADL-Barthel, Barthel Index for Activities of Daily Living; LEDD, the levodopa equivalent daily dose; Sti-on, Stimulation on; Sti-off, Stimulation off.

Values are shown as mean ± SD.

*: MoCA was evaluated in the on medication/on stimulation state. Not all patients undergo cognitive test at each follow-up due to the progression of PD that some patients are unable to cooperate in completing the examination.
